# Hydroxychloroquine, a successful treatment for lung disease in ABCA3 deficiency gene mutation: a case report

**DOI:** 10.1186/s13256-020-02604-5

**Published:** 2021-02-02

**Authors:** Waleed Shaaban, Majeda Hammoud, Ali Abdulraheem, Yasser Yahia Elsayed, Nawal Alkazemi

**Affiliations:** 1 Neonatology Department, Maternity Hospital, Kuwait, Kuwait; 2 Paediatrics Department, Faculty of Medicine, Kuwait University, Kuwait, Kuwait

**Keywords:** ABCA3, Pulmonary surfactant, Hydroxychloroquine, ChILD, Case report

## Abstract

**Background:**

Pulmonary surfactant is a complex mixture of lipids and specific proteins that stabilizes the alveoli at the end of expiration. Mutations in the gene coding for the triphosphate binding cassette transporter A3 (ABCA3), which facilitates the transfer of lipids to lamellar bodies, constitute the most frequent genetic cause of severe neonatal respiratory distress syndrome and chronic interstitial lung disease in children. Hydroxychloroquine can be used as an effective treatment for this rare severe condition.

**Case presentation:**

We report a late preterm Bosnian baby boy (36 weeks) who suffered from a severe form of respiratory distress syndrome with poor response to intensive conventional management and whole exome sequencing revealed homozygous ABCA3 mis-sense mutation. The baby showed remarkable improvement of the respiratory condition after the initiation of Hydroxychloroquine, Azithromycin and Corticosteroids with the continuation of Hydroxychloroquine as a monotherapy till after discharge from the hospital.

**Conclusion:**

Outcome in patients with ABCA3 mutations is variable ranging from severe irreversible respiratory failure in early infancy to chronic interstitial lung disease in childhood (ChILD) usually with the need for lung transplantation in many patients surviving this rare disorder. Hydroxychloroquine through its anti-inflammatory effects or alteration of intra-cellular metabolism may have an effect in treating cases of ABCA3 gene mutations.

## Background

Pulmonary surfactant is a surface tension reducing substance at the air fluid interface that prevents alveolar collapse at the end of expiration. It is produced by alveolar type II cells, stored in the lamellar bodies and is secreted by exocytosis into the alveolar lumen. After secretion, surfactant is recycled by alveolar type II cells or cleared by alveolar macrophages. This need the presence of specific receptors on their cell walls shared with receptors for interleukin(IL)-3and IL-5 to do their job [1–3].

Surfactant is a complex mixture of lipids, mainly phosphatidylcholine and surfactant proteins (SP-A, SP-B, SP-C and SP-D). ABCA3 is a phospholipid transporter protein critical for surfactant homeostasis, it is located on the limiting membrane of lamellar bodies and has a key role in the transport of phospholipids into lamellar bodies during the biosynthesis of surfactant by hydrolyzing Adenosine Tri-Phosphate (ATP) [1, 4, 5]. ABCA3 protein is coded by a single gene located on chromosome 16 and consists of 33 exons [6]. Mutations in the ABCA3 gene can result in fatal surfactant deficiency in term new-born infants and later, child interstitial lung disease (ChILD) if they survive the neonatal period [7].

## Case presentation

We report a case of late preterm Bosnian baby boy (36 weeks), first of a non-identical twin. The baby was a product of a vacuum delivery to a primi-gravida mother and non-consanguineous Bosnian parents. Pregnancy was uneventful and the mother received a complete course of ante-natal dexamethasone. At birth, the baby was given an Apgar score of 8 and 9 at 1 and 5 minutes respectively. He showed signs of mild respiratory distress in the form of tachypnea with mild subcostal retractions.

**Fig. 1:**
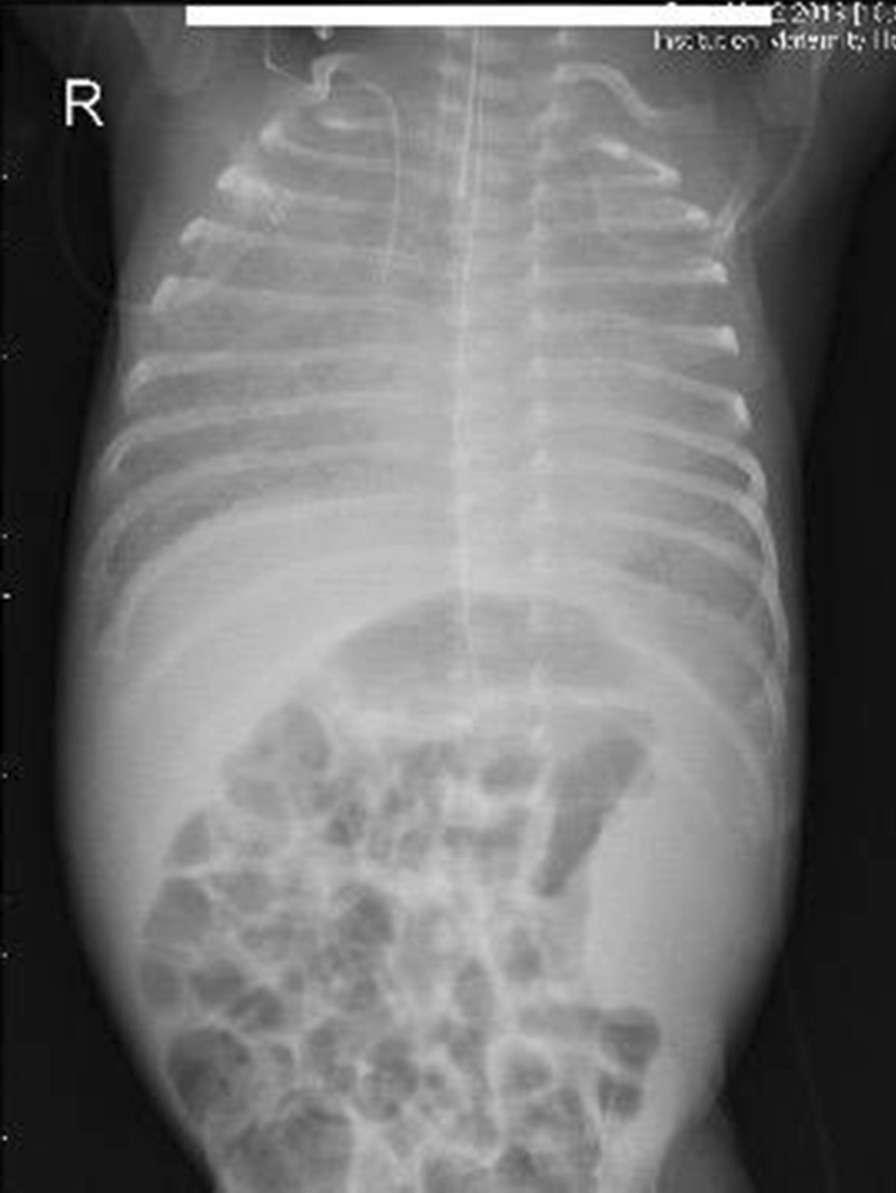
Patient’s chest X-ray on admission

**Fig. 2:**
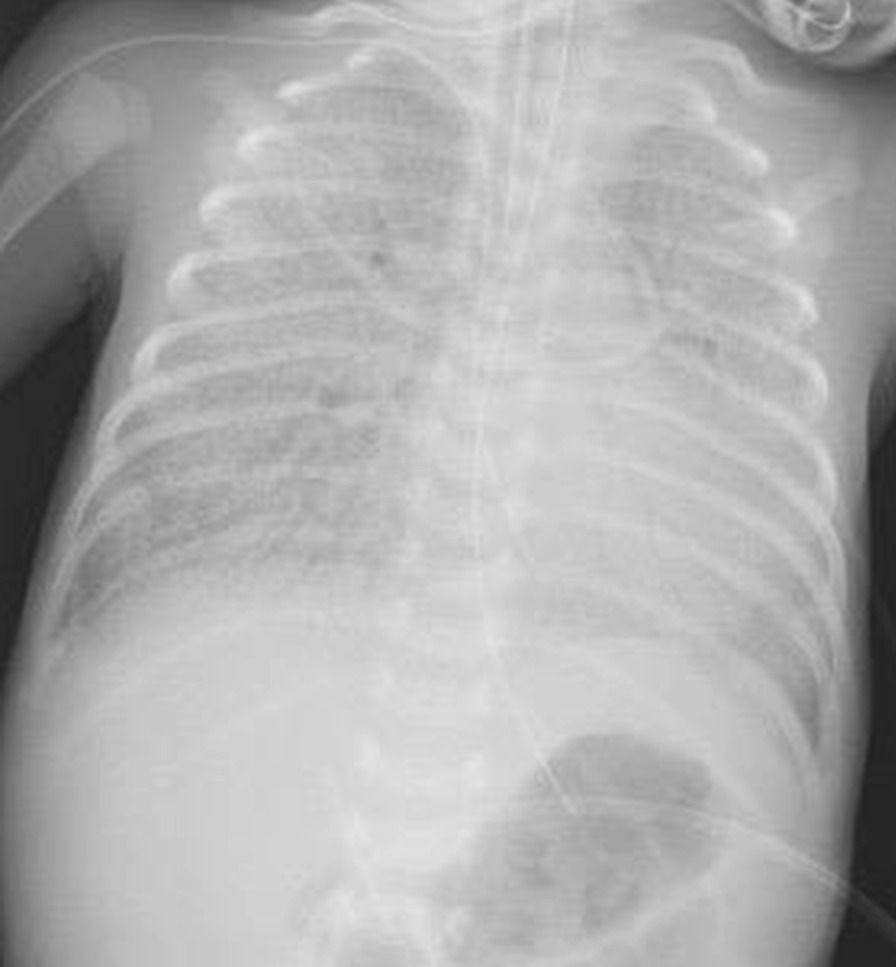
Patient’s chest X-ray during his early course of treatment

An immediate chest X-ray showed homogenous opacity of both lung fields disproportionate to his gestation (Fig. 1). The baby was initially managed by low flow nasal cannula which was replaced by high flow nasal cannula (HFNC) then NCPAP then the baby was intubated and connected to A/C mode of conventional ventilator, to end up on high frequency oscillation ventilation (HFOV) by the 3rd day of life due to the worsening of the respiratory distress, frequent desaturations and the chest X-ray which showed severe hyaline membrane disease (Fig. 2).

The baby received intra-tracheal surfactant during his stay in the NICU based on the chest X-ray findings of arterial blood gas values and the ventilatory parameters but with no improvement.

Other modalities of treatment were used including the following:

Plumozyme nebulization was tried according to the pulmonologist recommendations with no improvement so was stopped and continued with albuterol, ipratropium bromide nebulization and budesonide which was started and continued till the time of discharge. Diuretics (Furosemide and Aldactone) were started by day 14 of life with little benefits and then Furosemide was replaced by Hydrochlorothiazide with the continuation of Aldactone. An Echocardiogram done on day 6 of life showed ASD, large PDA and pulmonary hypertension. Both inhaled nitric oxide (continued till day 40 of life) and Sildenafil (continued till day 128 of life) were used to treat pulmonary hypertension. A 3-day course of Ibuprofen failed to close the PDA so it was surgically ligated on day 44 of life. Lung biopsy was not performed because of the clinical instability of the baby. High resolution computerized tomography (HRCT) was not done because of difficulty in mobilizing the child.

On day 52 of life, whole exome sequencing resulted in identifying a homozygous ABCA3 missense mutation c.875A < T and surfactant deficiency due to surfactant metabolism dysfunction was confirmed. Later both parents were found to be heterozygous carriers of the ABCA3 gene mutation using the same test.

On day 61 of life the following medications were started:

1. Hydroxychloroquine at a dose of 10 mg/kg/days and was planned to be continued as long as there was an improvement in the respiratory condition.
2. Methylprednisolone as a pulse therapy at a dose of 10 mg/kg/days for 3 doses and was planned to be repeated after one month but there was no need to repeat it as the baby improved.
3. Azithromycin 10 mg/kg/days for the first day followed by 5 mg/kg/days for the next 4 days.

**Fig. 3:**
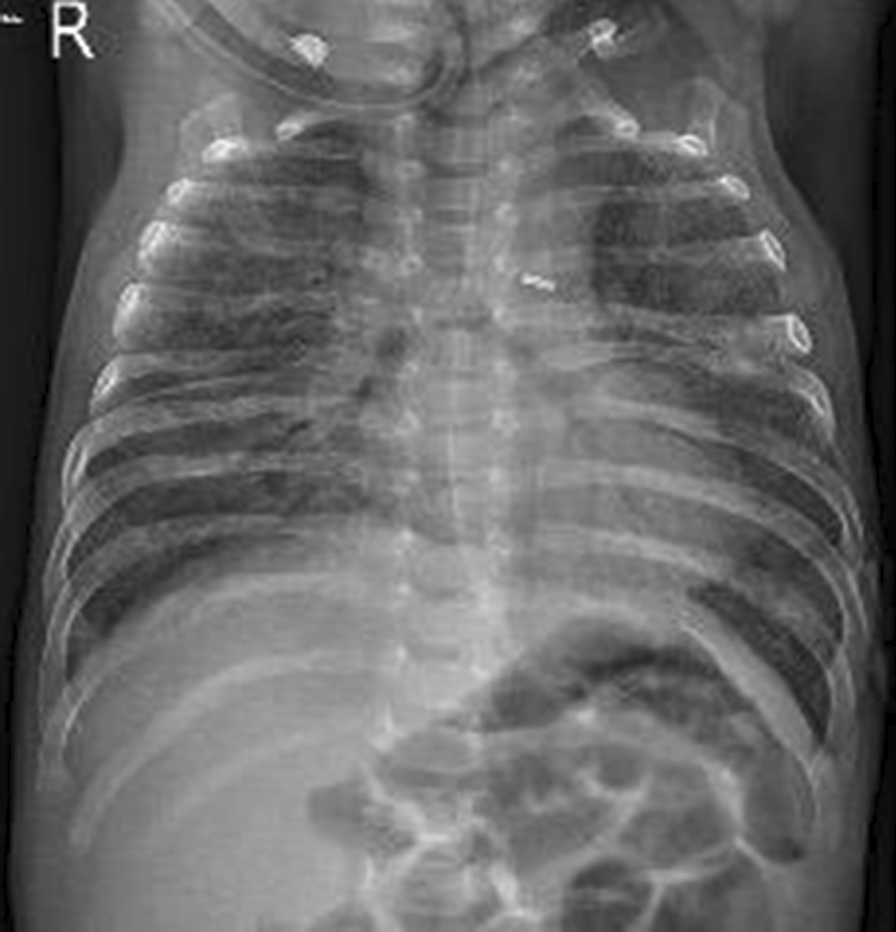
Patient’s chest X-ray after receiving hydroxychloroquine and just before discharge

After few days of starting this treatment, the baby started to show marginal improvement and we were able to gradually shift him from HFOV to A/C mode of conventional ventilation and then he was shifted to HFNC on day 107 of life guided by blood gases and the clinical condition of the baby. Chest X-ray also showed a remarkable improvement (Fig. 3).

Apart from the respiratory problem, the baby was always neurologically stable with normal cranial ultrasound, tolerating feeds well, did not have any hepatic, renal or hematological problems and had only once Gram-negative sepsis attack for which he received a 14-day course of Meropenem.

The baby was discharged home on day 160 of life in a good condition, breathing room air with good saturation and no respiratory distress. He is on exclusive breast feeding from his mother with good weight gain and on only Hydroxychloroquine as a maintenance therapy.

## Discussion and conclusions

Mutation in the gene encoding ATP binding cassette member A3 (ABCA3) can cause fatal respiratory distress in term infants [8, 9] and chronic interstitial lung disease in older children [10, 11].

ABCA3 gene mutation disturbs pulmonary surfactant metabolism through interfering with the importing of surfactant lipids into lamellar bodies. It is one of the surfactant dysfunction disorders (which also include SP-B and SP-C mutations) [12].

Histologically, with light microscopy, all surfactant dysfunction disorders share the same findings which are marked alveolar type II cells hyperplasia, interstitial thickening with mesenchymal cells which will end up with fibrosis. Macrophages and proteinaceous material may be present in air spaces. These findings are non-specific and may be present in any surfactant dysfunction disorder mutations [7, 8, 12].

Clinically, surfactant dysfunction disorders present usually with moderate to severe respiratory distress and signs of diffuse lung disease without satisfactory history or laboratory findings [13].

Diagnostic approaches include echocardiography, HRCT, bronchoscopy and broncho-alveolar lavage, lung biopsy with electron microscopy to differentiate between different forms of surfactant metabolism dysfunction, but a definitive diagnosis requires genetic studies [14, 15].

Therapeutic protocols with Hydroxychloroquine (HCQ), Corticosteroids and Macrolides in combination are suggested for patients with interstitial lung disease by SP-C deficiency and empirically used in patients with ABCB3 gene mutations [16].

The exact mechanism of action of HCQ is unknown but it may reduce interstitial inflammation and alter intracellular metabolism [17, 18]. Corticosteroids have been shown to up regulate the expression of ABCA3 in alveolar type II cells [19] and Macrolides can inhibit the production of many pro-inflammatory cytokines and inflammatory mediators involved in the development of interstitial fibrosis [20].

Duration of therapy is not clear in literature and there were reported cases of cure with no recurrences after withdrawal of HCQ in surfactant dysfunction deficiency cases [7, 16, 21].

Outcome in patients with ABCA3 mutations is variable, ranging from severe irreversible respiratory failure in early infancy to chronic static or progressive interstitial lung disease, with many patients surviving well into their second decade of life without lung transplantation [7].

In our case, HCQ, Corticosteroids and Azithromycin were used in combination with gradual improvement. Corticosteroids and Azithromycin were not continued as planned to get the benefits of monotherapy and to avoid the side effects of more than one drug.

The improvement of the chest condition in our case may be explained by the effect of the drugs with the aforementioned mechanisms, the increased expression of ABCA3 protein due to the age advancement, the presence of some normal lamellar bodies in ABCA3 deficient subjects, exposure to environmental stress or the variable natural history of the disease [7, 22].

The strong point of our work was the performance of parental whole exome sequencing which confirmed that the mutation was recessive in inheritance and not a uniparental disomy or de novo mutation. This helped us to inform the parents that they have a chance of 25% in each pregnancy to have a similar case and to guide them for pre-implantation embryo selection if they plan any future pregnancy.

## Data Availability

The data that supports the finding of this case report are available from the medical records of the neonatal department at Maternity Hospital, Kuwait.

## Abbreviations

ATP: Adenosine tri-phosphate
SP: Surfactant proteins
HCQ: Hydroxychloroquine
HFNC: High flow nasal cannula
A/C mode: Assist-control mode
HFOV: High frequency oscillation ventilation
ChILD: Child interstitial lung disease
NCPAP: Nasal continuous positive airway pressure
HRCT: High resolution computerized tomography
ASD: Atrial septal defect
PDA: Patent ductus arteriosus
